# Clinical relevance and outcome of familial papillary thyroid cancer: a single institution study of 626 familial cases

**DOI:** 10.3389/fendo.2023.1200855

**Published:** 2023-09-14

**Authors:** Zhuyao Li, Hongri Zhang, Yu Yan, Xiang Li, Meng Jia, Honglong Zhou, Xiubo Lu

**Affiliations:** ^1^ Department of Thyroid Surgery, The First Affiliated Hospital of Zhengzhou University, Henan, China; ^2^ Department of Neurosurgery, The First Affiliated Hospital, and College of Clinical Medicine of Henan University of Science and Technology, Henan, China; ^3^ School of Electrical and Information Engineering, Zhengzhou University, Henan, China; ^4^ Department of Urology Surgery, The First Affiliated Hospital of Zhengzhou University, Henan, China; ^5^ Department of Neurosurgery, The Second Affiliated Hospital of Nanchang University, Nanchang, China

**Keywords:** papillary thyroid cancer (PTC), familial, sporadic, prognosis, aggressive

## Abstract

**Background:**

Whether familial thyroid cancer is more aggressive than sporadic thyroid cancer remains controversial. Additionally, whether the number of affected family members affects the prognosis is unknown. This study focused mainly on the comparison of the clinicopathological characteristics and prognoses between papillary thyroid cancer (PTC) patients with and without family history.

**Methods:**

A total of 626 familial papillary thyroid cancer (FPTC) and 1252 sporadic papillary thyroid cancer (SPTC) patients were included in our study. The clinical information associated with FPTC and SPTC was recorded and analyzed by univariate analysis.

**Results:**

Patients in the FPTC group had a higher rate of multifocality (*p*=0.001), bilaterality (*p*=0.000), extrathyroidal invasion (*p*=0.000), distant metastasis (*p*=0.012), lymph node metastasis (*p*=0.000), recurrence (*p*=0.000), a larger tumor size (*p*=0.000) and more malignant lymph nodes involved (central: *p*=0.000; lateral: *p*=0.000). In addition, our subgroup analysis showed no significant difference (*p*>0.05) between patients with only one affected family member and those with two of more group in all clinicopathological characteristics. In papillary thyroid microcarcinoma (PTMC) subgroup analysis, we found that FPTMC patients harbored significantly larger tumors (*p*=0.000), higher rates of multifocality (*p*=0.014), bilaterality (*p*=0.000), distant metastasis (*p*=0.038), lymph node metastasis (*p*=0.003), greater numbers of malignant lymph nodes (central: *p*=0.002; lateral: *p*=0.044), higher rates of I-131 treatment (*p*=0.000) and recurrence (*p*=0.000) than SPTMC patients.

**Conclusion:**

Our results indicated that PTC and PTMC patients with a positive family history had more aggressive clinicopathological behaviors, suggesting that more vigilant screening and management for FPTC may be helpful.

## 1 Introduction

Thyroid cancer has increased substantially worldwide (1), driven largely by an increase in nonmedullary thyroid cancer (NMTC) (2). NMTC is prevalently sporadic (approximately 90%), but an increasing number of studies have reported that 3% to 9% are found with a family history (3, 4), named familial nonmedullary thyroid cancer (FNMTC). Papillary thyroid cancer (PTC) accounts the majority part of the FNMTCs according to the histological classification (approximately 85%), followed by follicular thyroid cancer (approximately 6%), anaplastic thyroid cancer (approximately 1.4%) and oncocytic (Hürthle) thyroid cancer (5).

With the increasing cases of thyroid cancer, it’s of great importance for clinicians to know whether familiar thyroid cancer is more aggressive than sporadic disease and whether the affected family numbers also have an effect on the disease. Studies showed that there would be a 5-9-fold increased risk of thyroid cancer for individuals with only one first-degree relative which was diagnosed with thyroid cancer (6). For those patients with two or more affected family members which diagnosed as thyroid cancer, the probability rises to 53-99% (7, 8). Unfortunately, the genetic reasons have not yet been characterized (9). Moreover, diagnosticians cannot distinguish sporadic from familial thyroid cancer by histology.

Some epidemiologic and clinical studies have reported that FNMTC has much more possibility to occur local invasion, tumor multifocality, lymph node metastasis and local or regional recurrence than SNMTC (10, 11). All these features suggest that clinicians use more vigilant screening and management in affected families. However, few studies focus in the specific pathological type and the sample size was relatively small in almost all the studies. Consequently, whether the PTC share exactly the same characteristics with NMTC in familial genetic aspect is still undetermined. This study focused on the comparison of the clinical characteristics and between FPTC and SPTC patients.

## 2 Patients and methods

### 2.1 Patients

**Figure 1:**
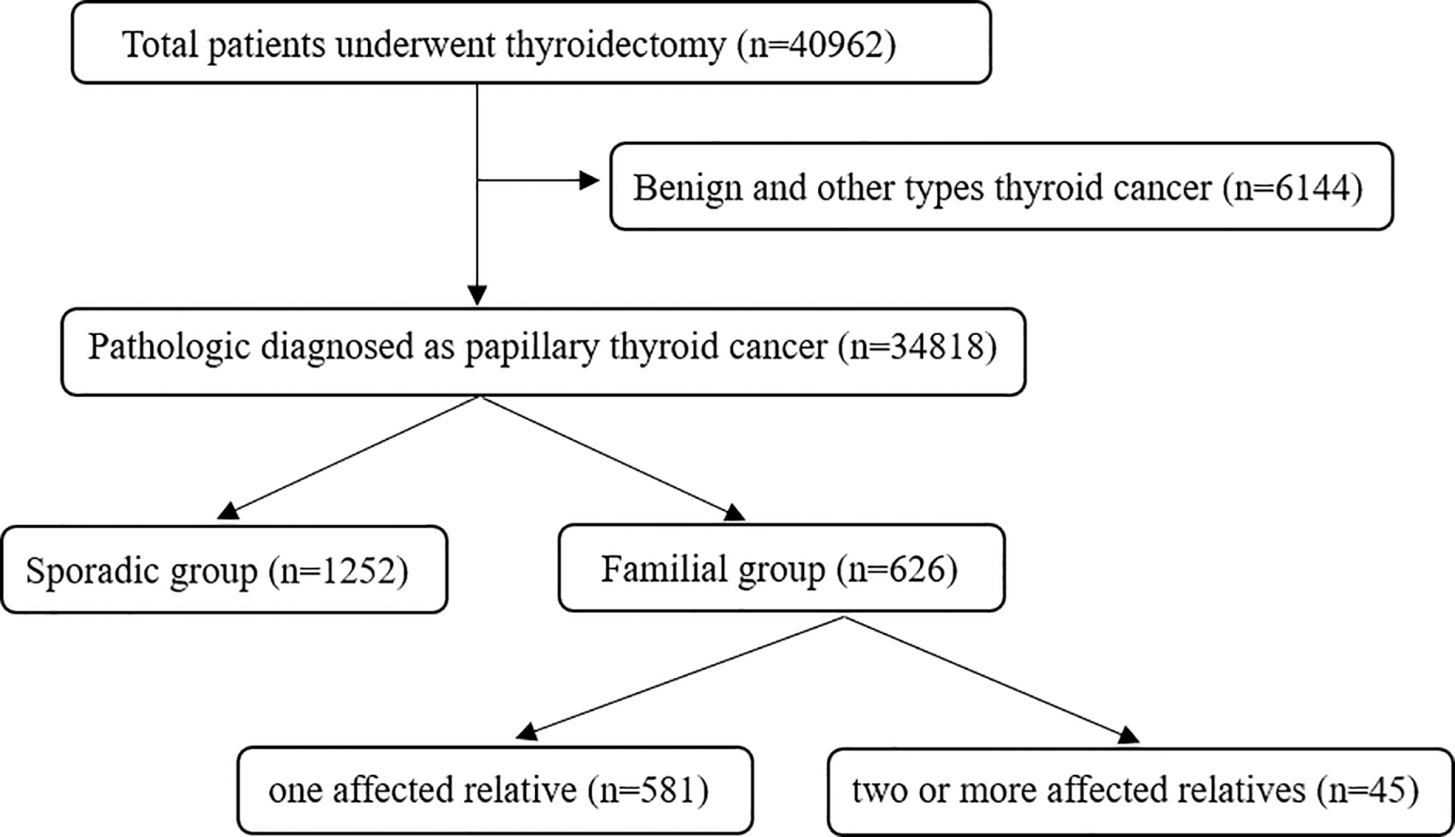
Study flow chart of familial papillary thyroid cancer patients.

A retrospective study was designed and carried out on the papillary thyroid cancer (PTC) patients who underwent thyroidectomy at The First Affiliated Hospital of Zhengzhou University from January 2012 to December 2021. The diagnosis of PTC was based on pathological results. All included patients were classified as having FPTC when at least one first-degree relative was diagnosed with PTC. In total, 626 PTC patients were classified as having FPTC, including 581 patients with one affected relative and 45 with two or more affected relatives. SPTC patients were randomly selected from 34,818 PTC patients during the same time (**Figure 1**). Random numbers were generated using Excel. The size of the SPTC patient sample was determined using a ratio of SPTC : FPTC=2:1. Patients who experienced neck surgery for other diseases or had a radiation explosion history were excluded. Papillary thyroid microcarcinoma (PTMC) was defined as the PTC with a diameter of no more than 10 mm. This study was approved by the Ethics Committee of The First Affiliated Hospital of Zhengzhou University, and informed consent was signed by all included patients.

### 2.2 Methods

Clinical information was recorded, including family history, age at diagnosis, lymph node metastasis, tumor size, TNM staging, I-131 treatment and operation information such as lymph node dissection, bilaterality, multicentricity, local invasion and extrathyroidal extension (**Tables 1**, **2**). The TNM stage was determined according to the American Joint Committee on Cancer (AJCC) (8th ed., 2017). Bilaterality was defined as PTC present in both thyroid lobes. Multifocality was diagnosed when two or more tumors were found in one or both lobes. Any extension exceeding the thyroid gland was defined as extrathyroidal extension.

**Table 1:** Clinical and pathological characteristics of patients.

	Overall			Subgroup		*p*	Statistical method
	Apparent familial	Sporadic	*p*	One affected member	Two or more affected members		
	(n=626)	(n=1252)		(n=581)	(n=45)		
Age at diagnosis	43.64 ± 11.3	44.64 ± 11.2	0.065	43.6 ± 11.4	43.7 ± 9.9	0.987	t-test
Sex (male/female)	160/466	282/970	0.144	150/431	10/35	0.594	χ^2^
Tumor size (cm)	1.05 ± 0.83	0.83 ± 0.65	0.000^*^	1.06 ± 0.83	0.96 ± 0.80	0.411	t-test
Multifocality			0.001^*^			0.989	χ^2^
Y	222 (35%)	351 (28%)		206 (35%)	16 (36%)		
N	404 (65%)	901 (72%)		375 (65%)	29 (64%)		
Bilaterality			0.000^*^			0.346	χ^2^
Y	184 (29%)	233 (19%)		168 (25%)	16 (36%)		
N	442 (71%)	1019 (81%)		413 (75%)	29 (64%)		
Extrathyroidal invasion			0.000^*^			0.881	χ^2^
Y	79 (13%)	72 (6%)		73 (13%)	6 (13%)		
N	547 (87%)	1180 (94%)		508 (87%)	39 (87%)		
Distant metastasis			0.012^*^			0.640	χ^2^/Fisher’s exact
Y	19 (3%)	17 (1%)		17 (3%)	2 (4%)		
N	607 (97%)	1235 (99%)		564 (97%)	43 (96%)		
TNM staging (2017)			0.652			0.338	Wilcoxon
I	593 (95%)	1179 (94%)		549 (95%)	44 (98%)		
II	24 (4%)	65 (5%)		23 (4%)	1 (2%)		
III	8 (1%)	7 (1%)		8 (1%)	0 (0%)		
IV	1 (0%)	1 (0%)		1 (0%)	0 (0%)		
Operation			0.000^*^			0.754	χ^2^
Total thyroidectomy	418 (67%)	568 (45%)		387 (67%)	31 (69%)		
Lobectomy	208 (33%)	684 (55%)		194 (33%)	14 (31%)		
Lymph node dissection							
Central	536	1035		499	37		
Lateral	121	95		110	11		
None	93	217		85	8		
Lymph node metastasis			0.000^*^			0.978	χ^2^
Y	277 (44%)	445 (35%)		257 (44%)	20 (44%)		
N	349 (56%)	807 (65%)		325 (56%)	25 (56%)		
Central lymph node metastasis			0.001^*^			0.229	χ^2^
Y	262 (42%)	423 (36%)		247 (43%)	15 (33%)		
N	364 (58%)	829 (64%)		334 (57%)	30 (67%)		
Number of malignant central lymph nodes	1.32 ± 2.4 (n=262)	0.96 ± 1.9 (n=423)	0.000^*^	1.33 ± 2.5	1.18 ± 2.3	0.679	t-test
Lateral lymph node metastasis			0.000^*^			0.143	χ^2^
Y	81 (13%)	78 (6%)		72 (12%)	9 (20%)		
N	545 (87%)	1174 (94%)		509 (88%)	36 (80%)		
Number of malignant lateral lymph nodes	0.62 ± 2.1 (n=81)	0.26 ± 1.4 (n=78)	0.000^*^	0.61 ± 2.2	0.76 ± 1.73	0.649	t-test
HT			0.428			0.689	χ^2^
Y	72 (12%)	160 (13%)		66 (11%)	6 (13%)		
N	554 (88%)	1092 (87%)		515 (89%)	39 (87%)		
I-131 treatment			0.000^*^			0.502	χ^2^
Y	168 (27%)	166 (13%)		154 (27%)	14 (31%)		
N	458 (73%)	1086 (87%)		427 (73%)	31 (69%)		
Recurrence			0.000^*^			0.411	χ^2^/Fisher’s exact
Y	38 (6%)	9 (1%)		34 (6%)	4 (9%)		
N	588 (94%)	1243 (99%)		547 (94%)	41 (91%)		
Follow-up (month)	45.1 ± 18.4	44.1 ± 13.6	0.156	44.7 ± 18.0	51.3 ± 23.0	0.020^*^	t-test

n, number; Y, yes; N, no; TNM, tumor, node, metastasis; HT, Hashimoto’s thyroiditis; * indicates statistical significance.

**Table 2:** Clinical and pathological characteristics of Apparent familial PTMC vs. Sporadic PTMC and Apparent familial PTC >1 cm.

Variables	Apparent familial PTMC (n=450)	Sporadic PTMC (n=948)	*p*	Apparent familial PTC >1 cm (n=176)	*p*	
Age at diagnosis	43.7 ± 11.0	44.7 ± 10.8	0.123	43.5 ± 12.1	0.864	t-test
Sex (male/female)	106/344	207/741	0.471	54/122	0.066	χ^2^
Tumor size (cm)	0.65 ± 0.27	0.57 ± 0.26	0.000^*^	2.1 ± 0.87	0.000^*^	t-test
Multifocality			0.014^*^		0.000^*^	χ^2^
Y	136 (30%)	228 (24%)		86 (49%)		
N	314 (70%)	720 (76%)		90 (51%)		
Bilaterality			0.000^*^		0.000^*^	χ^2^
Y	109 (24%)	136 (14%)		75 (43%)		
N	341 (76%)	812 (86%)		101 (57%)		
Extrathyroidal invasion			0.114		0.000^*^	χ^2^
Y	26 (6%)	37 (4%)		53 (30%)		
N	424 (94%)	911 (96%)		123 (70%)		
Distant metastasis			0.038^*^		0.016^*^	χ^2^/Fisher’s exact
Y	9 (2%)	7 (1%)		10 (6%)		
N	441 (98%)	941 (99%)		166 (94%)		
TNM staging (2017)			0.775		0.007^*^	Wilcoxon
I	433 (96%)	909 (96%)		160 (91%)		
II	14 (3%)	36 (4%)		10 (6%)		
III	2 (0.4%)	2 (0%)		6 (3%)		
IV	1 (0.2%)	1 (0%)		0 (0%)		
Operation			0.000^*^		0.000^*^	χ^2^
Total thyroidectomy	274 (61%)	364 (38%)		144 (82%)		
lobectomy	176 (39%)	584 (62%)		32 (18%)		
Lymph node dissection						
Central	380	766		156		
Lateral	59	48		62		
None	72	182		21		
Lymph node metastasis			0.003^*^		0.000^*^	χ^2^
Y	170 (38%)	282 (30%)		107 (61%)		
N	280 (62%)	666 (70%)		69 (39%)		
Central lymph node metastasis			0.002^*^		0.000^*^	χ^2^
Y	165 (37%)	270 (28%)		97 (55%)		
N	285 (63%)	678 (72%)		79 (45%)		
Numbers of malignant central lymph node	0.96 ± 1.7 (n=)	0.73 ± 1.6 (n=)	0.014^*^	2.2 ± 3.6	0.000^*^	t-test
Lateral lymph node metastasis			0.006^*^		0.000^*^	χ^2^
Y	35 (8%)	40 (4%)		46 (26%)		
N	415 (92%)	908 (96%)		130 (74%)		
Numbers of malignant lateral lymph node	0.33 ± 1.2 (n=)	0.18 ± 1.3 (n=)	0.044^*^	1.4 ± 3.4	0.000^*^	t-test
HT			0.660		0.493	χ^2^
Y	50 (11%)	113 (12%)		23 (13%)		
N	400 (89%)	835 (88%)		153 (87%)		
I-131 treatment			0.000^*^		0.000^*^	χ^2^
Y	79 (18%)	70 (7%)		89 (51%)		
N	371 (82%)	878 (93%)		87 (49%)		
Recurrence			0.000^*^		0.006^*^	χ^2^/Fisher’s exact
Y	20 (4%)	7 (1%)		18 (10%)		
N	430 (96%)	941 (99%)		158 (90%)		
Follow-up (month)	45.2 ± 18.2	44.2 ± 13.7	0.252	44.9 ± 18.9	0.844	t-test

PTMC, papillary thyroid microcarcinoma; PTC, papillary thyroid carcinoma; n, number; Y, yes; N, no; TNM, tumor, node, metastasis; HT, Hashimoto’s thyroiditis; * indicates statistical significance.

### 2.3 Operation

All surgical strategies in our study were based on the Chinese Thyroid Association clinical practice guidelines. Lobectomy was performed for single-lesion patients who had a tumor size less than 2 cm and no lymph node metastasis, extrathyroidal invasion or radiation explosion history. Total thyroidectomy was performed for patients with at least one of the following characteristics: 1. bilateral lesions, 2. multiple lesions, and 3. single lesions with a history of lymph node metastasis, extrathyroidal invasion or radiation explosion history. Prophylactic central compartment node dissection (CCND) was performed for all patients with a defined preoperative diagnosis of PTC through fine needle aspiration (FNA). Modified radical neck dissection (mRND) was performed on patients with lateral neck lymph node metastases. Additional I-131 treatment was performed for patients with extrathyroidal extension, lateral neck lymph node metastasis or tumor size >2.5 cm. The thyroid hormone suppressive therapy was performed to all patients in our study after the operation.

### 2.4 Postoperative follow-up

Postoperative follow-up items, including serum tests such as free triiodothyronine (FT3), free thyroxin (FT4), thyroid stimulating hormone (TSH), thyroid peroxidase antibody (TPOAb) and anti-thyroglobulin antibodies (TgAb) levels, ultrasound or positron emission tomography (PET)-CT, were performed on all included patients every 1-3 months within one and half years. During our follow-up time, abnormal serum Tg and TgAb levels or other clinical suspicions of recurrence, such as suspicious imaging findings (ultrasound, (PET)-CT, X-ray), were diagnosed as recurrence only when pathologically proven.

### 2.5 Statistical analysis

The results are presented as the mean ± SD for continuous variables and percentages for categorical variables. Chi-square tests and Fisher’s exact tests were used for continuous variables. Student’s t-test and Wilcoxon rank-sum test were used for categorical variables. All *p*-values were two-sided. A statistically significant difference was considered when the *p*-value<0.05. All analyses were performed using SPSS (Version 22.0, Chicago, IL, USA).

## 3 Results

### 3.1 Clinical characteristics

In total, 1878 patients were included in our study. Of these patients, 33.3% (626/1878) had FPTC, and the remaining 66.7% (1252/1878) had SPTC. The female-to-male ratios of FPTC and SPTC are 2.9 and 3.4, respectively. The mean ages of patients with FPTC and SPTC were 43.64 ± 11.3 (range from 10 to 75) and 44.64 ± 11.2 (range from 14 to 77), respectively. No significant differences were found between FPTC and SPTC in terms of sex (*p*=0.144) and age (*p*=0.065) or in all subgroup analyses.

### 3.2 Comparison between FPTC and SPTC groups

Statistical analysis showed that FPTC patients undertook more total thyroidectomy and I-131 treatments than SPTC patients (total thyroidectomy: 67% vs. 45%, *p*=0.000; 27% vs. 13%, *p*=0.000). Significant differences were found in terms of tumor size (1.05 ± 0.83 vs. 0.83 ± 0.65 cm; *p*=0.000), multifocality (35% vs. 28%; *p*=0.001), bilaterality (29% vs. 19%; *p*=0.000), extrathyroidal invasion (13% vs. 6%; *p*=0.000), distant metastasis (3% vs. 1%; *p*=0.012), lymph node metastasis (44% vs. 35%; *p*=0.000), number of malignant lymph nodes (central: 1.32 ± 2.4 vs. 0.96 ± 1.9, *p*=0.000; lateral: 0.62 ± 2.1 vs. 0.26 ± 1.4, *p*=0.000) and recurrence (6% vs. 1%, *p*=0.000) in the FPTC group than in the SPTC group. For the TNM stage, Hashimoto’s thyroiditis distribution and follow-up time, however, no statistically significant difference was found (TNM stage: *p*=0.652; Hashimoto’s thyroiditis: 12% vs. 13%; p=0.428; follow-up time: familial: 45.1 ± 18.4; sporadic: 44.1 ± 13.6).

### 3.3 Subgroup analysis according to affected family members

A further comparison was performed in the FPTC group between patients with one affected relative and those with two or more affected relatives. No significant difference was found in terms of age (43.6 ± 11.4 vs. 43.7 ± 9.9; *p*=0.987), sex (150/431 vs. 10/35; *p*=0.594), tumor size (1.06 ± 0.83 vs. 0.96 ± 0.80 cm; *p*=0.411), multifocality (35% vs. 36%; *p*=0.989), bilaterality (25% vs. 36%; *p*=0.346), extrathyroidal invasion (13% vs. 13%; *p*=0.881), lymph node metastasis (44% vs. 44%; *p*=0.978), distant metastasis (3% vs. 4%; *p*=0.640), and I-131 treatment (27% vs. 31%; *p*=0.502). In the treatment section, similarly, no significant difference was found between these two groups. In addition, there was no significant difference in recurrence between patients with one affected relative and those with two or more affected relatives (6% vs. 9%; *p*=0.411).

### 3.4 Subgroup analysis according to tumor diameter

The first subgroup comparison was performed between FPTMC and SPTMC. Compared to patients with SPTMC, patients with FPTMC had larger tumor sizes (0.65 ± 0.27 vs. 0.57 ± 0.26 cm; *p*=0.000) and higher rates of multifocality (30% vs. 24%; *p*=0.014), bilaterality (24% vs. 14%; *p*=0.000), distant metastasis (2% vs. 1%; *p*=0.038), lymph node metastasis (38% vs. 30%; *p*=0.003), numbers of malignant lymph nodes (central: 0.96 ± 1.7 vs. 0.73 ± 1.6, *p*=0.002; lateral: 0.33 ± 1.2 vs. 0.18 ± 1.3, *p*=0.044), I-131 treatment (18% vs. 7%; *p*=0.000) and recurrence (4% vs. 1%; *p*=0.000). In the operation section, FPTMC patients received a much higher rate of total thyroidectomy than those in the SPTMC group (61% vs. 38%; *p*=0.000); therefore, by contrast, people who received lobectomy only accounted for 39% in the FPTMC group, which was much less than that in the SPTMC group (39% vs. 62%; *p*=0.000). However, we failed to find significant differences in extrathyroidal invasion (6% vs. 4%; p=0.114), TNM staging (*p*=0.775) and Hashimoto’s thyroiditis (11% vs. 12%; p=0.660).

The second subgroup comparison was performed between patients in the FPTMC and FPTC >1 cm groups. Patients in the FPTC group with a tumor size larger than 1 cm had extremely higher rates of multifocality (30% vs. 49%; *p*=0.000), bilaterality (24% vs. 43%; *p*=0.000), extrathyroidal invasion (6% vs. 30%; p=0.000), distant metastasis (2% vs. 6%; *p*=0.016), lymph node metastasis (38% vs. 61%; *p*=0.000) and recurrence (4% vs. 10%; *p*=0.006) and greater numbers of malignant lymph nodes (central: 0.96 ± 1.7 vs. 2.2 ± 3.6, *p*=0.000; lateral: 0.33 ± 1.2 vs. 1.4 ± 3.4, *p*=0.000) than those observed in FPTMC patients. Logically, according to the larger tumor size, fewer people received total thyroidectomy in the FPTMC group than in the FPTC >1 cm group (61% vs. 82%; *p*=0.000), similar to the I-131 treatment (18% vs. 51%; *p*=0.000). In addition, we found that FPTMC patients had lower TNM stages than did patients in the FPTC >1 cm group (*p*=0.007). No significant difference was found with regard to Hashimoto’s thyroiditis (11% vs. 13%; p=0.493).

### 3.5 Comparison according to the new WHO standard

According to the 5^th^ edition (2022) of the WHO classification of endocrine and neuroendocrine tumors, a group of heterogeneous hereditary thyroid cancers which occur in families with follicular cell derived thyroid cancer as the major cancer is defined as Non-syndromic familial follicular cell-derived thyroid carcinoma (NSFNMTC), and should presence of follicular cell derived thyroid cancer in at least three first-degree relatives or papillary thyroid cancer in two or more first-degree relatives. Based on the new edition, there were 45 patients included in our data, then a comparison between NSFNMTC patients and sporadic patients was conducted presently (**Table 3**).

**Table 3:** Clinical and pathological characteristics in the new WHO standard.

	NSFNMTC	Sporadic	*p*	Statistical method
	(n=45)	(n=45)		
Age at diagnosis	43.7 ± 10.0	42.6 ± 9.0	0.383	t-test
Sex (male/female)	10/35	11/34	0.803	χ^2^
Tumor size (cm)	0.96 ± 0.80	0.35 ± 0.13	0.000^*^	t-test
Multifocality			0.006^*^	χ^2^
Y	16(36%)	5(11%)		
N	29(64%)	40(89%)		
Bilaterality			0.000^*^	χ^2^
Y	16(36%)	1(2%)		
N	29(64%)	44(98%)		
Extrathyroidal invasion			0.026^*^	χ^2^/Fisher’s exact
Y	6(13%)	0(0%)		
N	39(87%)	45(100%)		
Distant metastasis			1.000	χ^2^/Fisher’s exact
Y	2(4%)	1(2%)		
N	43(96%)	44(98%)		
TNM staging (2017)			0.317	Wilcoxon
I	44(98%)	45(100%)		
II	1(2%)	0(0%)		
III	0(0%)	0(0%)		
IV	0(0%)	0(0%)		
Operation			0.000^*^	χ^2^
Total thyroidectomy	31(69%)	2(4%)		
Lobectomy	14(31%)	43(96%)		
Lymph node dissection			0.000^*^	
Central	37	20		
Lateral	11	0		
None	8	25		
Lymph node metastasis			0.000^*^	χ^2^
Y	20(44%)	2(4%)		
N	25(56%)	43(96%)		
Central lymph node metastasis			0.000^*^	χ^2^
Y	15(33%)	1(2%)		
N	30(67%)	44(98%)		
Number of malignant central lymph nodes	1.2 ± 2.3 (n=14)	0.02 ± 0.1 (n=1)	0.000^*^	t-test
Lateral lymph node metastasis			0.003^*^	χ^2^/Fisher’s exact
Y	9(20%)	0(0%)		
N	36(80%)	45(100%)		
Number of malignant lateral lymph nodes	0.76 ± 1.7 (n=9)	0.0 ± 0.0 (n=0)	0.000^*^	t-test
HT			0.748	χ^2^
Y	6(13%)	5(11%)		
N	39(87%)	40(89%)		
I-131 treatment			0.000^*^	χ^2^
Y	14(31%)	0(0%)		
N	31(69%)	45(100%)		
Recurrence			0.242	χ^2^/Fisher’s exact
Y	3(7%)	0(0%)		
N	42(93%)	45(100%)		
Follow-up (month)	37.2 ± 23.2	36.9 ± 22.1	0.859	t-test

NSFNMTC, Non-syndromic familial follicular cell-derived thyroid carcinoma; n, number; Y, yes; N, no; TNM, tumor, node, metastasis; HT, Hashimoto’s thyroiditis; * indicates statistical significance.

The statistical analysis showed NSFNMTC patients have larger tumor sizes (0.96 ± 0.80 vs. 0.35 ± 0.13 cm; *p*=0.000) and higher lymph node metastasis rate (central: 33% vs. 2%; *p*=0.000; lateral: 20% vs. 0%; *p*=0.003) than sporadic patients. Moreover, significant differences were found in multifocality (36% vs. 11%; *p*=0.006), bilaterality (36% vs. 2%; *p*=0.000), extrathyroidal invasion (13% vs. 0%; *p*=0.026). Besides, comparing with the sporadic patients, NSFNMTC patients were more likely to choose total thyroidectomy (69% vs. 4%; *p*=0.000) and I-131 treatments (31% vs. 0%; *p*=0.000) since their higher rate of bilaterality and extrathyroidal invasion. While in distant metastasis and recurrence section, we did not find significant difference.

## 4 Discussion

Whether FPTC patients should be treated with a more aggressive surgical procedure is controversial (12). Several studies have compared the clinical characteristics of FNMTC with those of sporadic nonmedullary thyroid cancer (SNMTC) and concluded that there was no significant clinicopathological characteristic difference between these two groups (13, 14). However, the FNMTC patients in these studies included both PTC and anaplastic thyroid carcinoma (ATC). Given the prominent differences in clinical characteristics between ATC and PTC, the comparison results may not be the same as those for FPTC. Reports based solely on analyses of FPTC are few and limited by sample size. Sung and colleagues studied 238 FPTC patients and concluded that the multifocality and bilaterality rates in FPTC were higher than those in SPTC (42.4% vs. 33.9%; 26.9% vs. 20.2%, respectively) (15). Cao et al. included 372 familial PTC patients (16) and reported that there was a significant difference between FPTC and SPTC groups in recurrence-free survival (RFS) (7.31% vs. 1.30%). Furthermore, multivariate analysis showed that the presence of a family history was an independent risk factor for recurrence (*p*=0.004). Therefore, we conducted the largest retrospective study, to date, to analyze the differences in clinical characteristics, treatments and pathology results between the FPTC and SPTC groups and found that patients in the FPTC group had higher rates of larger tumor size, multifocality, bilaterality, extrathyroidal invasion and distant metastasis than those patients in the SPTC group. In addition, a significantly higher rate of recurrence was identified in FPTC group, indicating a more aggressive role of a positive family history.

Compared with other clinical characteristics, extrathyroidal invasion is one of the most important factors in making surgical plans. Our data showed that the extrathyroidal invasion rate in the FPTC group was significantly higher than that in the SPTC group (13% vs. 6%, *p*< 0.001), suggesting that more aggressive treatments, such as total thyroidectomy, lymph mode dissection and I-131 treatment, should be considered for FPTC patients. Seemly, Lei et al. recommended a more aggressive surgical strategy to obtain a better relapse-free survival for FPTC patients (17). This was consistent with other studies (14). Furthermore, lymph node metastasis is another important factor in judging the prognosis of patients, and studies have found that PTC patients with cervical lymph node metastasis have a 1.32-fold higher risk of having an adverse prognosis than those without (*p*=0.011) (18). Yu et al. (19) similarly suggested that lymph node metastasis can decrease patients’ overall survival, as analyzed through Kaplan–Meier curves; in addition, the Cox model indicated that lymph node metastasis contributed a 1.36 hazard ratio (HR) and consequently could be used as a predictor for adverse prognosis in PTMC patients as well. Regarding our data, since there were only a few cases of recurrence in our study, we did not use a Cox model to analyze the effect of lymph node metastasis on recurrence. However, we observed a significantly higher rate of lymph node metastasis in the FPTC group than in the SPTC group (44% vs. 35%, *p*< 0.001), suggesting an adverse prognosis in patients with a positive family history. This finding was also supported by Zhang’s study (14) in 2016 (52.6% vs. 33.3%, *p*= 0.001).

Benign thyroid diseases mainly include Hashimoto’s thyroiditis (HT), hyperthyroidism, hypothyroidism, subacute thyroiditis and thyroid follicular nodular disease, among which HT is the most common human autoimmune disease, with a prevalence of more than 5% in females. A long-term retrospective study (20) indicated that the rate of patients with PTC combined with HT in the FNMTC group was higher than that in the SNMTC group (30.6% vs. 23.0%, *p*< 0.05). Our data failed to reveal a significant difference between FPTC and SPTC with respect to the HT rate (12% vs. 13%, *p*= 0.428). Additionally, the subgroup analysis did not find a significant difference in HT distribution between FPTC patients with one affected relative and FPTC patients with two or more affected family members (11.4% vs. 13.3%, *p* = 0.689).

The incidence of PTMC has increased in recent years (21), and 74% (1398/1878) of cases were PTMC in the current study. However, the therapy for PTMC patients is still controversial. Some studies have suggested that PTMC should be treated with moderate therapy but that more attention should be paid to PTMC patients with affected family members since they have a less aggressive disease course; indeed, more attention should be paid to PTMC patients with a positive family history. Cao et al. (16) carried out Kaplan–Meier survival analyses and concluded that FPTMC patients had a worse RFS than SPTMC patients (*p* = 0.002). Sung’s study (15) showed that FPTMC patients had higher rates of multiple clinicopathological features, such as multifocality (*p* = 0.044), extrathyroidal invasion (*p* = 0.000), and central lymph node metastasis (*p* = 0.006). Similarly, our data also found adverse clinical pathologic characteristics and prognoses in FPTMC patients compared with SPTMC patients. However, FPTMC patients showed a less aggressive disease course than SPTMC patients. Therefore, we suggest that a positive family history does have an adverse effect on both PTC and PTMC patients. In summary, we suggest a more invasive therapy, such as prophylactic central lymph node dissection and additional I-131 treatment, for FPTMC patients as well. This conclusion was also supported by a recent study (22).

The definition of FNMTC was indeterminacy due to the lack of a specific laboratory test before the 5^th^ edition of WHO published. Chinese experts indicated that non-medullary thyroid carcinoma patients who have two or more first relatives diagnosed as NMTC, or with one NMTC first relative and at least three relatives with thyroid follicular nodular disease can be defined as family non-medullary thyroid carcinoma. While American surgeons defined FNMTC as two or more first-degree relatives existing in one family without exposure history related to thyroid cancer or other familial syndrome (23). Furthermore, the links between clinicopathological characteristics and the numbers of family members affected by FPTC are still controversial either. Lakis et al. (10) indicated that FNMTC patients with two or more affected family members had a higher rate of lymph node metastasis than those with only one affected relative (*p*=0.008). Triponez et al. (24) found that comparing with those who have two affected relative member, FNMTC patients with three or more affected members had a significantly shorter survival time. However, other studies have reported the opposite result; for example, Lee et al. (25) found the affected family members has no effect on the clinicopathological characteristics of FNMTC patients. Hillenbrand’s study (26) showed that FNMTC patients with three affected family members shared a comparable tumor size with patients who had two affected relatives (*p*=0.644). Additionally, our data showed that there was no difference in clinicopathological characteristics and recurrence rates between FPTC patients with one and two or more affected family numbers (15, 25, 27).

After the 5^th^ edition of WHO classification of endocrine and neuroendocrine tumors published, to further identifying whether the stricter diagnostic criteria would affect the outcome, we carried out a comparison between the NSFNMTC patients and sporadic patients. Interestingly, it almost identical to the results of previous analysis between FPTC and SPTC patients in aspects of larger tumor size, multifocality, bilaterality, extrathyroidal invasion and lymph node metastasis. NSFNMTC patients possess a higher rate of total thyroidectomy and undertook more I-131 treatment than those sporadic patients. As regard to distant metastasis and recurrence, which performs opposite to other characteristics, supposing due to the small sample capacity, showed no statistical differences. Furthermore, although shared similar results with the first comparison, we found a greater distance between NSFNMTC with the sporadic group and FPTC with the control. It may be inferred that under the stricter classification, NSFNMTC patients might harbor more aggressive clinicopathological behaviors than FPTC patients comparing with the sporadic group.

Nixon et al. indicated that only when at least two first degree relatives were diagnosed could decrease the sporadic event to less than 5%, then the “true” FNMTC diagnosis could be confirmed (28). It cannot be denied that the diagnosis of NSFNMTC should be defined as existing two or more first-degree relatives diagnosed as PTC to exclude the possibility of randomness. It might present some minor problems, however, the first two family members could not be identified as FNMTC correctly (28), accordingly, the treatment strategy might have something different. Early detection and treatment of diseases are of great significance, our data indicated that PTC patients with positive family history had more aggressive clinicopathological behavior than sporadic PTC patients. In China or other developing countries, some people do not have awareness to get the annual examination at their whole life, they went to the hospital only when they feel something different such as trachyphonia, dysphagia or others, which is absolutely late for thyroid disease. Therefore, we would recommend patients who have positive family history to suggest their family members pay attention to their thyroid during our clinical work. Thanks to the advice, some patients received treatment timely. Consequently, we recommend that a warning should be established once a patient have one first-degree relative.

The genetic background of syndromic familial follicular cell-derived thyroid carcinoma (SFNMTC) has been well established, but for the non-syndromic familial follicular cell-derived thyroid carcinoma (NSFNMTC) the heredity predisposing alterations has not been identified yet. Nevertheless, genetic factors have been widely reported as an important contributor to FPTC (9). Bann et al. identified a novel Y1203H germline Dual Oxidase-2 (DUOX2) mutation from the data of whole-exome sequencing of an FNMTC kindred (29). DUOX2 is enzymatically active and could increase the production of reactive oxygen species, suggesting the dysregulation of proteins involved in H2O2 metabolism may be the mechanism underlying genetic factors that increase thyroid cancer susceptibility (29). Bonora et al. reported two novel variants respectively in exon 9 and exon 13 of TIMM44 though the systematic screening of 14 candidate genes mapping to the region of linkage in affected TCO members in eight families with thyroid oncocytic tumors (30). The author inferred that any impairment in TIMM44 functions might be related to oncocytic proliferation (30). The role of the abovementioned genes in tumorigenesis may partly explain the more aggressive disease course in PTC patients with a positive family history.

Our study has several strengths in analyzing a family history effect on PTC patients. Age and sex have been reported as risk factors for thyroid cancer (19). The current study also took these two factors into consideration and found no difference between the FPTC and SPTC groups, which partly decreased the bias in analyzing the effect of a positive family history on the clinicopathogenetic characteristics of PTC patients. Therefore, there is still one shortcoming in our study. Some patients underwent their first operations in 2018, there were 31 patients with follow-up times of less than 24 months (22.7 ± 1.4 months), and they accounted for barely 1.7% of the subjects in our study. In consideration of the relatively nice prognosis of papillary thyroid carcinoma patients, no death event was observed during the follow-up period of this study and the recurrence cases were small as well, we did not focus on the survival and prognosis aspects but on the clinical and pathological characteristics areas.

## 5 Conclusions

In conclusion, our results indicated that familial PTC had a more aggressive clinicopathological behavior than sporadic PTC. Considering the relatively higher rates of lymph note metastasis and recurrence in FPTC patients, more invasive surgical treatments and I-131 treatments might be recommended to achieve a better relapse-free survival. Additionally, people with first-degree relatives diagnosed with PTC should alert the potential “anticipation” of thyroid cancer.

## Data availability statement

The raw data supporting the conclusions of this article will be made available by the authors, without undue reservation.

## Ethics statement

Written informed consent was obtained from the individual(s), and minor(s)’ legal guardian/next of kin, for the publication of any potentially identifiable images or data included in this article.

## Author contributions

ZL contributed to the conception and design of this study. HRZ contributed to the comparison according to the new WHO standard. XL was responsible for the collection of all data in this research. YY and MJ completed the statistical analysis. The final manuscript drafting and revising were completed by MJ, HLZ and XBL. All authors contributed to the article and approved the submitted version.
